# The Role of Physical Exercise to Improve the Browning of White Adipose Tissue via POMC Neurons

**DOI:** 10.3389/fncel.2018.00088

**Published:** 2018-03-28

**Authors:** Kellen C. da Cruz Rodrigues, Rodrigo M. Pereira, Thaís D. P. de Campos, Rodrigo F. de Moura, Adelino S. R. da Silva, Dennys E. Cintra, Eduardo R. Ropelle, José R. Pauli, Michel B. de Araújo, Leandro P. de Moura

**Affiliations:** ^1^Laboratory of Molecular Biology of Exercise, School of Applied Sciences, University of Campinas, Limeira, Brazil; ^2^Department of Health Science, Federal University of Lavras, Lavras, Brazil; ^3^School of Physical Education and Sport of Ribeirão Preto, University of São Paulo, Ribeirão Preto, Brazil; ^4^Laboratory of Nutritional Genomics, School of Applied Sciences, University of Campinas, Limeira, Brazil; ^5^Center of Research in Sport Sciences, School of Applied Sciences, University of Campinas, Limeira, Brazil; ^6^University Catholic Center of Quixadá (Unicatolica), Quixadá, Brazil; ^7^Postgraduate Program in Motricity Sciences, São Paulo State University, São Paulo, Brazil

**Keywords:** WAT browning, physical exercise, hypothalamus, POMC, AgRP

## Abstract

Obesity is a public health issue that affects more than 600 million adults worldwide. The disease is characterized by fat accumulation, mainly in the abdominal area. The human body is mainly composed of two types of adipose tissue: white adipose tissue (WAT) and brown adipose tissue (BAT); however, the browning process generates a different type of brown fat-like adipocyte in WAT, which similar to BAT has thermogenic capacity by activating UCP-1. The hypothalamic arcuate nucleus plays an important role in WAT browning via POMC neurons, which are influenced by synergistic insulin and leptin signaling. On the other hand, stimulation of AgRP neurons suppresses WAT browning. The hypothalamic inflammatory process that occurs in obesity impairs insulin and leptin signaling in this tissue and, consequently, can decrease WAT browning. In addition, practicing physical exercise may be a great strategy for triggering the browning process since it reduces hypothalamic inflammation and increases POMC neurons gene expression. Moreover, physical exercise stimulates irisin gene expression, which has an important impact on thermogenesis, which in turn culminates in increased gene expression of proteins such as UCP-1 and Cidea, which are related to WAT browning. Furthermore, thermogenetic activation of WAT leads to increased energy expenditure, favoring obesity treatment. Therefore, this mini-review aimed to highlight the most recent studies that link the control of hypothalamic activity with the browning metabolism of adipose tissue in response to physical exercise.

## Introduction

Nowadays, obesity can be considered a pandemic disease that affects more than 600 million adults worldwide (World Health Organization, 2016). The human body is composed mainly of two types of adipose tissue: White Adipose Tissue (WAT) and Brown Adipose Tissue (BAT). These tissues have different gene expressions, morphological distributions, and functions in the body (Dodd et al., 2015; Gómez-Hernández et al., 2016). While WAT stores energy and releases hormones and cytokines that regulate metabolism and insulin resistance, BAT expends energy to produce heat through non-shivering thermogenesis, via mitochondrial uncoupling protein 1 (UCP-1), as an adaption process to cold exposure (Dodd et al., 2015; Bargut et al., 2016).

The WAT browning process generates a different type of brown fat-like adipocyte in WAT, which has been called “recruitable brown fat cells,” “beige cells,” “adaptive brown fat cells,” or “brite cells”(Enerbäck, 2009; Ishibashi and Seale, 2010; Petrovic et al., 2010), and even though these cells do not have the same origin as BAT adipocytes, they have thermogenic capacity by activating UCP-1 (Bargut et al., 2016; Flouris et al., 2017). Although beige adipocytes are found interspersed in WAT, the browning process occurs primarily in subcutaneous WAT (Stanford et al., 2015). Moreover, beige adipocytes differ from WAT because they have a multilocular morphology and express UCP-1 as well as the cells expressing unique gene markers such as Tbx1, Tmem26, and Cd137, which are not expressed by both mature white and brown adipocytes (Stanford et al., 2015).

The central nervous system (CNS) plays a key role in WAT browning regulation. Specific neuronal populations such as agouti-related protein (AgRP) neurons and pro-opiomelanocortin (POMC) perform opposing functions to regulate WAT browning (Ruan et al., 2014; Dodd et al., 2015). While co-infusion of insulin and leptin activates POMC neurons and increases WAT browning (Dodd et al., 2015), fasting and chemical or genetic activation of AgRP neurons suppresses WAT browning (Ruan et al., 2014).

In WAT, the browning process and consequently thermogenesis can be stimulated by external factors such as cold exposure, medication, and physical exercise (Contreras et al., 2016). Therefore, therapies aiming to activate thermogenesis in WAT might be a great strategy to treat and prevent obesity (Wu et al., 2014; Nakhuda et al., 2016; Flouris et al., 2017). Practicing physical exercise may trigger the browning process since it reduces hypothalamic inflammation and increases POMC neuron gene expression (Chiarreotto-Ropelle et al., 2013; Laing et al., 2016). Moreover, physical exercise stimulates irisin gene expression, which has an important impact on thermogenesis (Boström et al., 2012; Chiarreotto-Ropelle et al., 2013). Therefore, this mini-review aimed to describe the mechanisms by which physical exercise regulates WAT browning through POMC neurons.

### The hypothalamus and its role in WAT browning via POMC and AgRP neurons

In the hypothalamus, two populations of neurons found in the arcuate nucleus stand out (Contreras et al., 2016). Orexigenic neurons are composed of NPY and AgRP and their activation increases food intake and reduces energy expenditure. On the other hand, when anorexigenic neurons (POMC and cocaine and amphetamine regulated transcript—CART) are activated, food intake is decreased, while energy expenditure is increased (Toda et al., 2017).

### POMC neuron activation stimulates WAT browning

POMC neurons seem to be the key point in thermogenesis-induced CNS, since they might activate BAT and stimulate WAT browning through the SNS (Zhu et al., 2016). Brown adipocyte thermogenic activity might be enhanced when noradrenaline is released, because cyclic adenosine monophosphate (cAMP) levels increase, which in turn activates protein kinase A (PKA) and the ultimate generation (through lipolysis) of fatty acids that are an energy substrate and UCP-1 activators (Labbé et al., 2015). Moreover, POMC neurons release α-melanocyte-stimulating hormone (α-MSH), which binds to MC4R—the main receptor of the melanocortin system involved in energy homeostasis (Labbé et al., 2015). Several brain populations of MC4R expressing-neurons are (poly) synaptically connected to BAT, thus highlighting the relevance of the melanocortin system in the metabolic control of BAT activity (Labbé et al., 2015). On the other hand, WAT cells that show beige activation, innervation, and propagation of the SNS signal are sparse, thus cell-to-cell communication plays a key role in the browning process (Zhu et al., 2016). Zhu et al. (2016) demonstrated the importance of the gap junction connexin 43 (Cx43) protein in WAT browning when they blocked Cx43 channels of connexin protein subunits and observed decreases in POMC-activation-induced adipose tissue browning.

Initially, POMC neurons were considered a homogeneous population that responded in a similar way to the actions of hormones and nutrients. However, several studies have demonstrated the heterogeneity of this population, and it has been shown that peripheral hormones such as insulin and leptin, which are related to energy expenditure control, work in different POMC populations (Williams et al., 2010; Dodd et al., 2015; Toda et al., 2017). Furthermore, these hormones have a synergistic effect on WAT browning via CNS.

**Figure 1:**
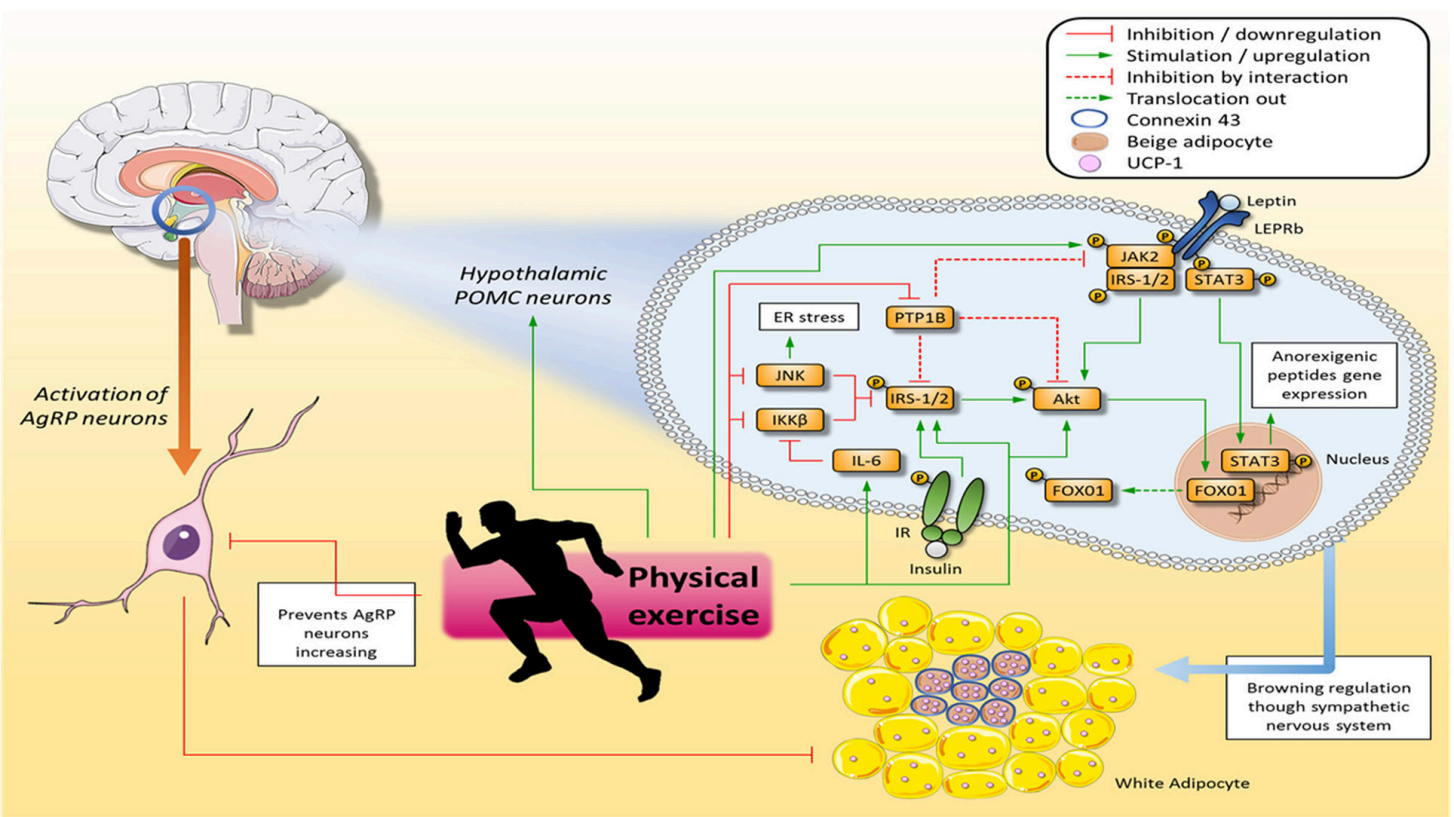
Physical exercise ameliorates hypothalamic insulin and leptin signaling, and consequently improves diet control and WAT browning and stimulates thermogenesis. Physical exercise improves leptin signaling in POMC neurons by increasing JAK2 and STAT3 tyrosine phosphorylation, which in turn migrates to the nucleus and transcribes anorexigenic neuropeptides. Furthermore, physical exercise enhances IRS-1/2 and Akt activation, which in turn improves Fox01 phosphorylation, allowing this protein to translocate out of the nucleus and stop transcribing orexigenic neuropeptides. Finally, proteins that inhibit insulin and leptin pathways, such as JNK, IKKβ, and PTP1B, have their activity decreased by physical exercise. Once insulin and leptin signaling has been restored in POMC neurons, the WAT browning process is increased via the sympathetic nervous system, and the signal propagation from an adipocyte receiving a sympathetic signal from a cluster of adipocytes in close proximity is connexin 43 (Cx43)-dependent. On the other hand, stimulation of AgRP neurons suppresses WAT browning. Akt, Protein kinase B; ER, Endoplasmic reticulum; FOX01, Forkhead box protein 01; IKKβ, Inhibitor of nuclear factor kappa-B kinase beta; IL-6, Interleukin 6; IR, Insulin receptor; IRS-1/2, Insulin Receptor Substrate 1 and 2; JAK2, Janus kinase 2; JNK, C-Jun-N terminal kinase; POMC, Pro-opiomelanocortin; PTP1B, Protein tyrosine phosphatase 1B; STAT3, Signal transducer and activator of transcription 3; WAT, White Adipose Tissue.

A study conducted by Dodd et al. (2015) demonstrated that insulin and leptin act synergistically to activate WAT browning through POMC neurons and this activation is at least partially dependent on phospoinositide 3-kinase (PI3K) to depolarize these neurons. To demonstrate this synergistic activation, the authors used double knockout (DKO) mice for the protein tyrosine phosphatase 1 B (PTP1B) and T-cell protein tyrosine phosphatase (TCPTP), which regulate the hypothalamic signaling of leptin and insulin, respectively. The authors observed that the body weight, body fat, and food intake of the animals decreased, although their ambulatory activity was not altered. On the other hand, the authors demonstrated that these results were related to thermogenesis and browning, since there was a significant increase in the gene expression of the browning-related proteins such as UCP-1, Prdm 16, cell death-inducing DFFA-like effector a (Cidea), Tmem26, and Cd137. In addition, the authors administered insulin, leptin, and a combination of these hormones via intracerebroventricular (ICV) injection and observed that the combined infusion potentiated thermogenesis in WAT. Furthermore, to show that WAT browning is stimulated via the autonomic nervous system, the authors denerved DKO mice that gained body weight and body fat as well as displaying WAT browning and BAT thermogenesis attenuation. In addition, Laing et al. (2016) demonstrated that chronic voluntary running wheel exercise (12 weeks) reduces high-fat diet-induced apoptosis in POMC expressing neurons in the hypothalamus. Moreover, Ropelle et al. (2010) showed that swimming and treadmill exercise restored the mRNA levels of POMC neurons diminished in diet-induced obese mice and in leptin deficient (ob/ob) mice (Figure 1).

### AgRP neuron activation suppresses WAT browning

Unlike POMC, AgRP neurons activation suppresses WAT browning and this mechanism is regulated by the hypothalamus (Ruan et al., 2014). Ruan et al. (2014) showed that fasting and AgRP activation are capable of reducing WAT browning mediated by O-linked β- N-acetylglucosamine (O-GlcNAc) transferase (OGT), which regulates important cellular processes of cytoplasmic and nuclear proteins and is increased in AgRP neurons in a fasting state. To demonstrate the role of AgRP neurons in reducing WAT browning the authors knocked out OGT in those neurons and observed a rise in WAT browning markers such as Cidea, Prdm16, and UCP-1 mRNA levels. Moreover, the ablation of OGT in AgRP neurons protects mice against diet-induced obesity and insulin resistance (Ruan et al., 2014). In addition, physical exercise might prevent AgRP neurons increasing in obese rats (Dragano et al., 2017) (Figure 1).

## Physical exercise improves insulin and leptin signaling in the brain

As previously described, insulin and leptin signaling play an important role in food intake and energy expenditure by regulating orexigenic and anorexic neurons and activating thermogenesis through POMC neurons. On the other hand, the combination of a high-caloric food intake and a sedentary lifestyle has been suggested as the most important etiological cause of obesity (Seaman, 2013), and this environment favors dysregulation of energy homeostasis, since diet-induced hypothalamic inflammation has been indicated as the earliest factor leading to insulin and leptin resistance in the hypothalamus (Dragano et al., 2017). In addition, regular physical exercise seems to be a possible way of ameliorating insulin and leptin signaling by regulating several proteins involved in their signal transduction pathways in the hypothalamus and altering the levels of orexigenic and anorexigenic neuropeptides (Benite-Ribeiro et al., 2016). Moreover, in a study that evaluated voluntary wheel running activity for 12 weeks, the authors demonstrated that physical activity was efficient in improving leptin signaling in the arcuate nucleus and in restoring the number of POMC neurons that were reduced in animals fed on a high fat diet (HFD) (Laing et al., 2016).

Inflammation processes impair insulin and leptin signaling in the hypothalamus and, consequently, decrease WAT browning; however, physical exercise has demonstrated its potent anti-inflammatory properties in this tissue. Chiarreotto-Ropelle et al. (2013) showed that acute exercise was efficient in reducing hypothalamic inflammation and PTP1B activity, which is increased when insulin and leptin resistance are installed. The mechanism proposed by the authors is that physical exercise disrupted the interaction between PTP1B and Insulin Receptor β (IRβ) and Insulin Receptor Substrate 1 (IRS-1) and Janus kinase 2 (JAK2), which are early proteins in insulin and leptin signaling, respectively. Moreover, physical exercise increased tyrosine phosphorylation in these proteins and reestablished the anorexic effects of insulin and leptin in obese rats.

In addition, physical exercise demonstrated its anti-inflammatory characteristics in another study with obese rats, in which the authors showed that inhibitor of nuclear factor kappa-B kinase beta (IKKβ) activation and endoplasmic reticulum (ER) stress seem to be the link between hypothalamic inflammation and hypothalamic insulin and leptin signaling impairment. In response to swimming exercise, the hypothalamic levels of Interleukin 6 (IL-6) in the rats increased, and IKKβ activation and ER stress were reverted. Moreover, physical exercise also restored hypothalamic insulin and leptin signaling, since the exercised obese rats showed an increase in the leptin-induced JAK2, IRS-1, Insulin Receptor Substrate 2 (IRS-2) and Signal transducer and activator of transcription 3 (STAT3) tyrosine phosphorylation, the insulin-induced IRβ, IRS-1, and IRS-2 tyrosine phosphorylation, as well as Akt and Fox01 serine phosphorylation (Ropelle et al., 2010). When phosphorylated by Protein kinase B (Akt), Forkhead box protein 01 (Fox01) leaves the nucleus and stops transcribing orexigenic neuropeptide genes (Rodrigues et al., 2015) (Figure 1). Furthermore, Yi et al. (2012) demonstrated that regular moderate exercise was effective in preventing and reverting hypothalamic inflammation caused by chronic consumption of a western diet.

Despite the benefits of physical exercise in reducing hypothalamic inflammation, which might improve WAT browning and its thermogenesis, it is important to highlight that the energy expenditure promoted by practicing physical exercise might often increase energy intake to achieve energy homeostasis. Furthermore, a recent study showed a positive correlation between rate of perceived exertion (RPE) and energy intake in healthy (not obese) children after practicing imposed exercise (Fearnbach et al., 2017). However, the effects of physical exercise in reducing energy intake depend on the metabolic status, since several studies are showing that only obese people decrease caloric intake as a consequence of practicing physical exercise practice (Fearnbach et al., 2015; Schwartz et al., 2016; Thivel et al., 2016) (Figure 1).

## Irisin: a potential link between physical exercise and WAT browning

Another protein that might be related to WAT browning through CNS is irisin, since it may alter orexigenic and anorexigenic neuropeptides (Ferrante et al., 2016). Irisin was initially described as a hormone-like myokine release from skeletal muscle after exercise stimulation that could up regulate Peroxisome proliferator-activated receptor-gamma co-activator 1-alpha (PGC-1α), a transcriptional co-activator related to energy metabolism (Boström et al., 2012). Studies of animals have demonstrated that physical exercise stimulates PGC1-α gene expression, which controls mitochondrial biogenesis and is a co-activator of Peroxisome proliferator-activated receptor gamma (PPAR-γ), which in turn modulates UCP-1 expression (Boström et al., 2012; Wrann et al., 2013). Moreover, PGC1-α stimulates the Fibronectin type III domain-containing protein 5 (FNDC5) gene expression that encodes irisin. To demonstrate the role of irisin in exercise-induced WAT browning, Boström et al. (2012) intraperitoneally injected anti-FNDC5 antibody into mice before they started a swimming training protocol lasting 10 days. The authors observed an abrupt reduction in physical exercise-induced UCP-1 and Cidea gene expressions in WAT, indicating that irisin is essential for WAT browning.

Physical exercise might activate thermogenesis by increasing FNDC5 / irisin gene expression, which is a protein that is able to increase UCP-1 mitochondrial expression in WAT, increasing the consumption of energy reserves and thermogenesis (Bonfante et al., 2017). On the other hand, FNDC5/irisin levels are diminished in people with obesity, hepatic steatosis, metabolic syndrome, and type 2 diabetes mellitus (Bonfante et al., 2017). Irisin is the secreted form of FNDC5 protein and has been proposed as the link between physical exercise and energy homeostasis. This hormone is secreted by both WAT and BAT, and it is positively correlated with leptin levels. Similarly to leptin, irisin seems to act in the CNS, including the hypothalamus, and plays an important role in food intake and energy expenditure (Ferrante et al., 2016). To demonstrate the role of irisin in the control of food intake, Ferrante et al. (2016) intrahypothalamically injected irisin (50 and 200 nmol/L) into rats and observed a reduction in food intake 24 h after infusion. In addition, the authors also demonstrated that the gene expressions of POMC and CART neurons were increased, while orexin-A was inhibited after irisin injection (Ferrante et al., 2016). Moreover, irisin was also identified in the human hypothalamus and cerebrospinal fluid (Piya et al., 2014) and this might be evidence that irisin produced in peripheral tissues acts in the CNS.

The role of physical exercise in increasing irisin levels and the WAT browning phenotype was also demonstrated by Brenmoehl et al. (2017). In this study the authors used the Dummerstorf marathon mouse model DUhTP, which has been selected over 90 generations for high treadmill performance, and they observed that the browning of WAT in DUhTP mice showed increased levels of T-box transcription factor (Tbx1), Peroxisome proliferator-activated receptor alpha (PPARα), UCP1, and heat production. Moreover, the authors observed improvements in oral glucose tolerance after 43 days, and together these results shed light on the role of physical exercise in ameliorating physiological parameters and increasing energy expenditure, which in turn might be a great strategy for treating and preventing obesity.

The role of physical exercise in increasing irisin levels and browning phenotypes has also been demonstrated in humans. Boström et al. (2012) observed an increase in serum irisin in eight male non-diabetic subjects after 10 weeks of aerobic training when compared to basal levels. In addition, using mass spectrometry, Jedrychowski et al. (2015) corroborated these findings when they observed that sedentary individuals had around 3.6 ng/mL of circulating irisin and after these individuals followed a 12-weeks high-intensity aerobic training protocol this level was increased to approximately 4.3 ng/ml (Boström et al., 2012). Furthermore, a meta-analysis has shown that a single bout of acute exercise is capable to immediately increasing irisin (Fox et al., 2017).

A study conducted by Bonfante et al. (2017) evaluated irisin levels in obese men who practiced combined training (CT) or remained sedentary (CG) for 24 weeks. The results showed that the CT group maintained their irisin levels, while the expression of this protein was reduced in the CG group. Besides preventing a decrease in irisin levels, chronic CT improved some metabolic parameters such as total cholesterol, LDL cholesterol, glucose, insulin, and the homeostatic model assessment of insulin resistance (HOMA-IR), reinforcing the importance of physical exercise in treating obesity (Bonfante et al., 2017). Moreover, Zhao et al. (2017) demonstrated that 12 weeks of resistance training were efficient in increasing the serum levels of irisin and reducing the fat percentage in older male adults.

## Irisin and exercise: tissue dependent results

Even though there is evidence that physical exercise might increase irisin levels and is associated with UCP1 up regulation and browning activation, some studies have not corroborated these findings (Qiu et al., 2015; Vosselman et al., 2015; Dinas et al., 2017): however they have important methodological problems. A systematic review conducted by Dinas et al. (2017) indicated several methodological limitations in the articles evaluated, such as the quantification of irisin and consequently PGC-1α. To analyze irisin, they used ELISA, a non-validated method for analyzing circulating irisin content, since ELISA uses polyclonal antibodies, which increases the chance of unspecific binds. The gold standard for analyzing irisin is mass spectrometry. Regarding PGC-1α, the methodology in these articles was considered weak because they quantify the mRNA levels of PGC-1α, but not the protein content (Dinas et al., 2017). A meta-analysis with eight randomized controlled trials evaluated the effects of chronic exercise training on circulating levels of irisin in adults and the authors concluded that chronic physical exercise might reduce irisin circulating levels; however, they emphasize a lack of appropriated controls in several parameters that could alter irisin content, such as diet, volume, intensity, and type of exercise (Qiu et al., 2015).

A key point when evaluating the effects of physical exercise on thermogenesis is tissue specificity. A recent study showed that physical exercise plays antagonist roles in BAT and in subcutaneous WAT (scWAT), since it decreases BAT activation in core regions such as aortic BAT (aBAT) and interscapular BAT (iBAT) and increases browning in scWAT (Wu et al., 2014). Furthermore, in the article, Wu and colleagues induced obesity via a high fat diet (HFD) in rats and they also observed that endurance-trained obese animals showed no change in muscle FNDC5 content nor circulating irisin; however, the rats showed an increase in FNDC5 content in scWAT. Finally, the authors were able to show that physical exercise was efficient in increasing whole-body energy expenditure during the dark cycle, despite a thermogenesis reduction in classic BAT (Wu et al., 2014).

## Conclusions

In conclusion, practicing physical exercise may be considered an essential non-pharmacological strategy for fighting obesity. Among its beneficial effects, physical exercise may revert and prevent hypothalamic inflammation and, consequently, improve hypothalamic insulin and leptin signaling, which will control energy expenditure, hunger, and satiety processes. Since physical exercise improves the hypothalamic signaling of these hormones, it may also contribute to WAT browning, thereby increasing energy expenditure and preventing diet-induced obesity. Moreover, physical exercise might restore the mRNA levels of POMC neurons in obese mice, which may be a great strategy for increasing energy expenditure by activating WAT browning through POMC neurons. In addition, physical exercise stimulates an increase in serum irisin levels, which is essential for increasing proteins such as UCP-1 and Cidea, which are related to WAT browning.

## Author contributions

All authors contributed to the design of the article; KC and LM organized the article; KC wrote the article with support from TC and MA; RP, RM, AS, DC, ER, JP, and LM reviewed the manuscript.

### Conflict of interest statement

The authors declare that the research was conducted in the absence of any commercial or financial relationships that could be construed as a potential conflict of interest.
